# Magnetic Fields Affect Alcoholic Liver Disease by Liver Cell Oxidative Stress and Proliferation Regulation

**DOI:** 10.34133/research.0097

**Published:** 2023-03-30

**Authors:** Chao Song, Hanxiao Chen, Biao Yu, Lei Zhang, Junjun Wang, Chuanlin Feng, Xingxing Yang, Xiaofei Tian, Yixiang Fan, Xinmiao Ji, Hua Wang, Can Xie, Xin Zhang

**Affiliations:** ^1^ High Magnetic Field Laboratory, CAS Key Laboratory of High Magnetic Field and Ion Beam Physical Biology, HFIPS, Hefei, Anhui 230031, P.R. China.; ^2^ Science Island Branch of Graduate School, University of Science and Technology of China, Hefei, Anhui 230036, P.R. China.; ^3^ Institutes of Physical Science and Information Technology, Anhui University, Hefei, Anhui 230601, P.R. China.; ^4^ Department of Oncology, The First Affiliated Hospital of Anhui Medical University, Hefei, Anhui 230022, P.R. China.; ^5^ International Magnetobiology Frontier Research Center, Science Island, Hefei, Anhui 230031, P.R. China.

## Abstract

It is well known that alcohol consumption leads to substantially increased free radical levels and health risks, which lacks effective treatment besides alcohol abstinence. Here, we compared different static magnetic field (SMF) settings and found that a downward quasi-uniform SMF of ~0.1 to 0.2 T could effectively alleviate alcohol-induced liver damage and lipid accumulation and improve hepatic function. SMFs of two different directions can reduce the inflammation, reactive oxygen species levels, and oxidative stress in the liver, while the downward SMF had more obvious effects. Moreover, we found that the upward direction SMF of ~0.1 to 0.2 T could inhibit DNA synthesis and regeneration in hepatocytes, which caused detrimental effects on the lifespan of "heavy drinking" mice. In contrast, the downward SMF prolongs survival of "heavy drinking" mice. On one hand, our study shows that ~0.1 to 0.2 T moderate quasi-uniform SMFs with a downward direction have great promises to be developed into a physical method to reduce alcohol-induced liver damage; on the other hand, although the internationally recognized upper limit for SMF public exposure is 0.4 T, people should also pay extra attention to SMF strength, direction, and inhomogeneity that could generate harmful effects on specific severe pathological conditions.

## Introduction

Alcoholic liver disease (ALD) caused by excessive drinking is one of the most common chronic liver diseases, including steatosis, steatohepatitis, fibrosis, cirrhosis, and hepatocellular carcinoma [1]. ALD with a high short-term mortality has become a severe health threat and global burden [2–6], especially in current coronavirus disease 2019 pandemic situation [7,8]. A recent survey in about 7,528 alcoholics reveals that 27% of them have liver steatosis, 20% have steatohepatitis, and 26% have cirrhosis [9]. In fact, around 2 million people die from liver disease worldwide each year, and 50% of them have advanced liver cirrhosis [10]. However, although corticosteroids (prednisolone and prednisone) and pentoxifylline can be used to increase short-term survival of severe alcoholic hepatitis [11], there is no US Food and Drug Administration-approved drugs for ALD prevention or treatment besides abstinence from alcohol and subsequently nutritional supplements. Therefore, physicians are still actively seeking effective and safe therapies for the large number of patients with ALD.

Increased free radical level and oxidative stress in liver cells play a central role in the development of ALD [12–14]. Magnetic field can affect the spin state of electrons, providing a noninvasive physical method to manipulate the unpaired electrons in free radicals, which provides a theoretical basis for cellular reactive oxygen species (ROS) regulation by externally applied magnetic field [15–17]. In fact, it has been shown in multiple studies that static magnetic fields (SMFs) can affect ROS levels in many cells and animals, but the effects are variable [18]. Van Huizen et al. [19] showed that the ROS levels can be affected by SMFs in a field-intensity-dependent way, and Gurhan et al. [20] confirmed that different directions had various effects on ROS change, mitochondrial calcium, and cell growth in fibrosarcoma cell HT-1080. Moreover, our previous study shows that SMFs can affect ROS levels in cell-type-dependent manner [21]. Therefore, the effects of SMFs on ROS levels in biological systems are closely associated with magnetic field intensity, direction, and biological samples examined.

In recent years, electromagnetic fields have been applied as noninvasive physical tools to treat cancer, depression, and diabetes, including tumor-treating field, transcranial magnetic stimulation, and static magnetic and electric fields. For example, Carter et al. [22] recently show that a 3 mT SMF in combination of an electric field can treat type 2 diabetes through ROS and redox state regulation. Our previous study shows that a downward SMF of ~0.1 T can reduce ROS levels in pancreatic cells and alleviate type 2 diabetes [23]. Van Huizen et al. [19] show that the ROS levels in *Planaria* regeneration can also be decreased by 200 mT SMF to alter stem-cell-mediated growth. In addition, Yu et al. [24] found that SMF combined with the hybrid core–shell vesicles could increase ROS levels and promote ferroptosis-like cancer cell death. Therefore, modulating ROS and oxidative stress by electromagnetic field could potentially provide a noninvasive physical tool to regulate physiological and pathological processes.

In this study, we sought to examine the effects of SMFs in alcohol-induced liver damage using different mouse models and SMF settings. We found that for both shorter-term lighter drinking mice and longer-term heavier drinking mice, the alcohol-induced liver damage can be obviously relieved by a downward SMF of ~0.1 to 0.2 T. The lifespan of the "heavy drinking" mice was significantly prolonged by this magnetic field exposure. In contrast, a different SMF setting did not have such effects and even be detrimental to "heavy drinking" mice.

## Results

### The downward SMF alleviates alcohol-induced mouse liver damage

**Fig. 1.**
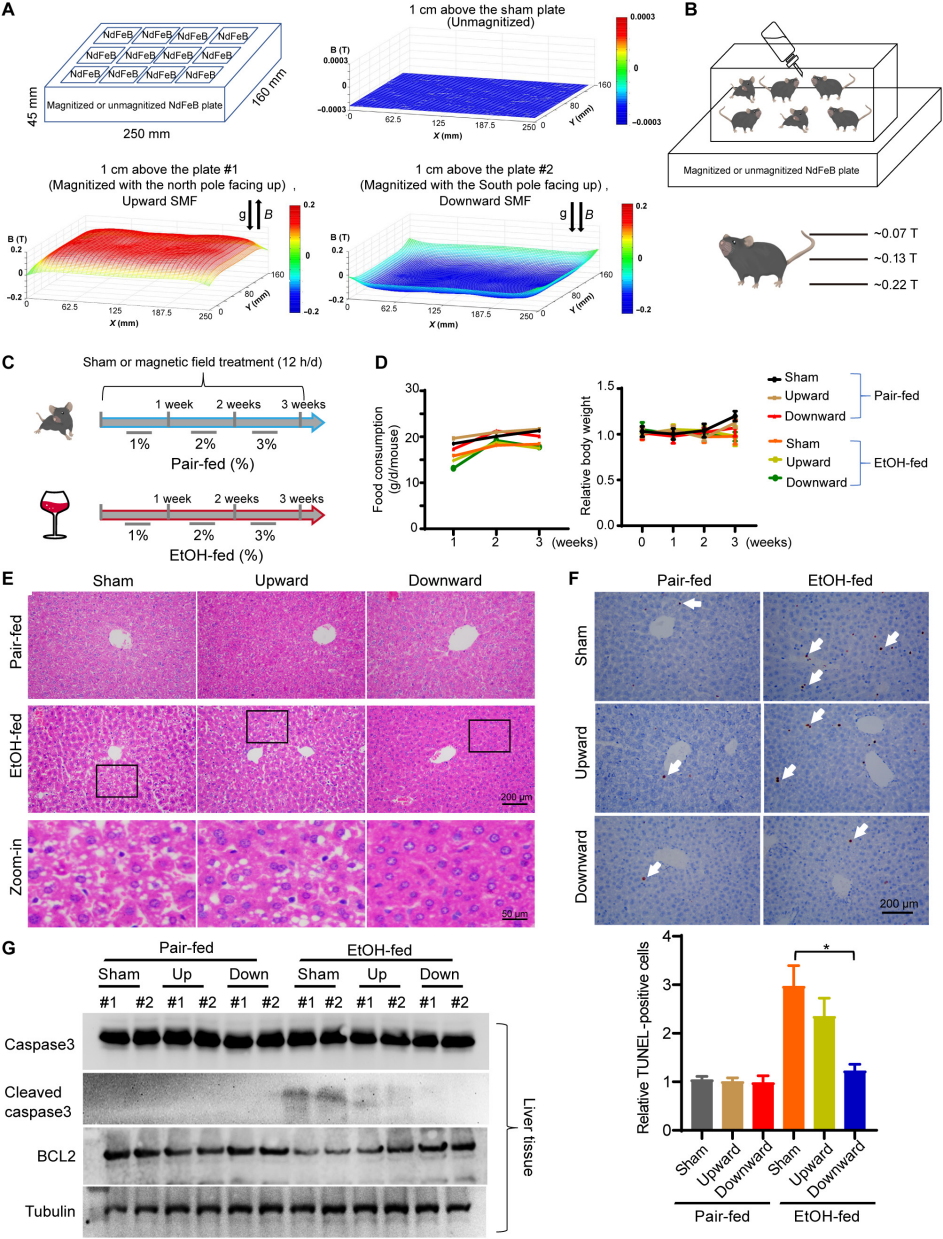
The downward static magnetic field alleviates alcohol-induced mouse liver damage. (A) Unmagnetized or magnetized NdFeB plates were used to provide sham, upward, or downward SMFs. Magnetic induction was measured at 1 cm above the plates with a magnet analyzer. (B) The whole mouse cage was placed on the magnetic plate, and the SMF on mice was ~0.1 to 0.2 T. (C) The mice were fed with unlimited access to the Lieber–DeCarli alcohol liquid diet containing either EtOH-fed or pair-fed with exposure to sham, upward, or downward SMFs for 3 weeks. (D) Food consumption and body weight were recorded every week. (E) Liver sections were subjected to H&E staining. (F) Liver section TUNEL assay and its quantification. Arrows indicate positively stained cells. *n* = 3 to 5 per group. (G) The protein levels of cleaved caspase 3, caspase 3, BCL2, and tubulin were detected by Western blot. All values represent means ± SEM. *P < 0.05 by Student's *t* test.

To assess the effects of SMF on alcohol-induced ALD, we used magnetic plates with different settings. The magnetic plates were composed of 12 closely connected N38 neodymium (NdFeB) magnet cubes, with either the north or south pole facing up, which provided a quasi-uniform magnetic flux density at a given horizontal plane (Fig. 1A). To reduce any potential placebo effects, we used unmagnetized NdFeB as sham control. We measured the average magnetic flux density at 1 cm above these quasi-uniform magnetic plates, which corresponds to the height of the mouse torso when they stand. The average magnetic flux densities of the 3 groups (sham, upward, and downward) at this horizontal level are 0.5, 128.5, and 134.5 mT, respectively. In addition, for these 250 mm (*L*) × 160 mm (*W*) magnetic plates, the magnetic flux is ~1.18 × 10^−4^, 4.62 × 10^−3^, and −4.50 × 10^−3^ Wb, respectively (Fig. 1A). The whole mouse cages were placed on the top of these plates (Fig. 1B), and the magnetic flux densities on the mice exposed to the magnetized plates were ~70 mT (at the mouse back) to 220 mT (at the mouse feet) (Fig. 1B). Most of the mouse bodies are exposed to 0.1 to 0.2 T SMFs.

We fed the mice with the Lieber–DeCarli alcohol liquid diet [25] containing either ethanol (EtOH-fed) or maltodextrin diet as a control (pair-fed) with unlimited access, a standard procedure for alcohol diet studies in the literature (Fig. 1C). For the EtOH-fed group, we gradually increased EtOH from 1% (week 1) to 2% (week 2) to 3% (week 3). We started the alcohol feeding and SMF exposure simultaneously from the beginning and continued for 3 weeks (SMF exposure was 12 h/d, 7 d/week) (Fig. 1C). We monitored their body weight, food consumption, and vital signs. No significant differences in body weight or food consumption were observed among EtOH-fed groups (Fig. 1D). For their vital signs, it seems that 3 weeks of alcohol consumption reduced both the heart rate (from 178.2 to 160.7, *P* < 0.05) and the arterial O_2_ (from 97.6% to 93.96%, *P* < 0.05), but SMFs can increase the mouse arterial O_2_ (Fig. S1).

Next, hematoxylin–eosin (H&E) staining was used to analyze the mouse tissue. No significant difference was observed in the spleen, lung, kidney, or heart (Fig. S2). However, all EtOH-fed mice had liver damage, which appeared as a large number of vacuoles on the liver sections. It is interesting that although both the upward and downward SMFs reduced the alcohol-induced vacuoles, especially the downward SMF, the upward SMF effects were not that obvious (Fig. 1E). Similarly, although both the upward and downward SMFs reduced the EtOH-induced liver cell apoptosis, the downward SMF had a much more significant alleviation effects as shown by the terminal deoxynucleotidyl transferase-mediated deoxyuridine triphosphate nick end labeling (TUNEL) assay (Fig. 1F). Western blot analysis also shows that the alcohol-induced cleaved caspase 3 was reduced by SMF treatment, especially by the downward SMF. The alcohol-induced B-cell lymphoma-2 (BCL2) reduction was also reversed by SMF treatment, especially by the downward SMF (Fig. 1G).

### The downward SMF alleviates alcohol-induced liver function and lipid metabolism abnormalities

**Fig. 2.**
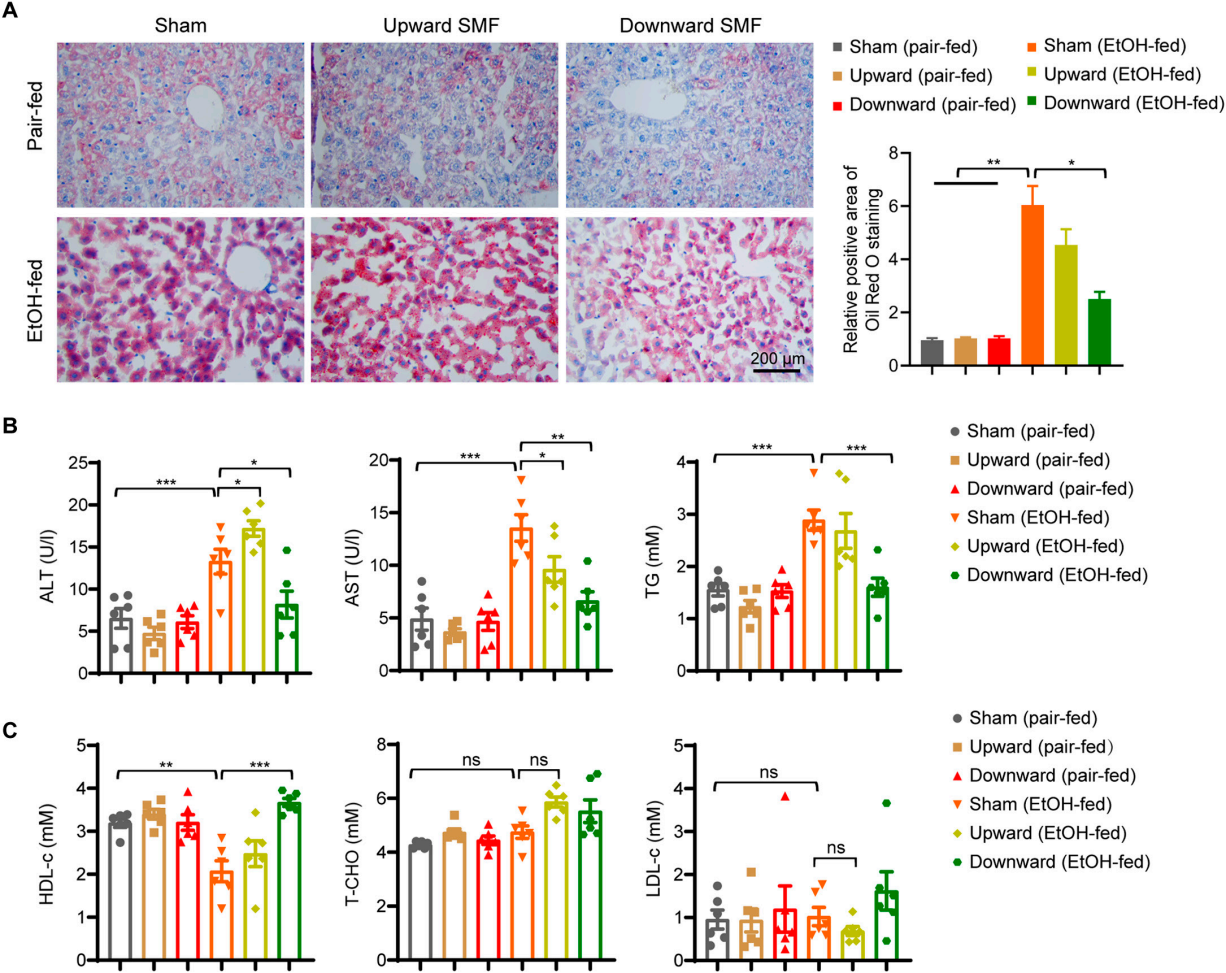
The downward static magnetic field alleviates alcoholic fatty liver. (A) Paraffin-embedded liver sections were used to assess lipid accumulation by Oil Red O staining and quantitatively analyzed. Scale bar, 200 μm. (B) Serum ALT, AST, and TG were measured. (C) Serum HDL-c, low-density lipoprotein cholesterol (LDL-c), and total cholesterol (T-CHO) were measured. Values represent means ± SEM, *n*=6 per group. *P < 0.05; **P < 0.01; ***P < 0.001 by Student's *t* test.

Since alcohol is known to perturb lipid metabolism, we next evaluate the effects of SMFs on alcohol-induced fatty liver, a common symptom for patients with ALD. We firstly used Oil Red O to stain fat deposition in liver tissue. It was found that the downward SMF significantly reduced lipid accumulation in the liver of EtOH-fed mice, while the upward SMF had no obvious effects (Fig. 2A). Moreover, we also measured multiple lipid indicators in the blood test that reflect the liver functions and lipid metabolism. We found that the alcohol-induced serum alanine aminotransferase (ALT), aspartate aminotransferase (AST), triglyceride (TG) elevation, and high-density lipoprotein cholesterol (HDL-c) reduction were all significantly alleviated by the downward SMF treatment but not the upward SMF (Fig. 2B and C). It should be mentioned that the HDL-c, which functions by removing excess cholesterol, was significantly reduced by the alcohol diet but can be reversed by the downward SMF. In contrast, the total cholesterol or low-density lipoprotein cholesterol was not much affected by the alcohol diet, and SMF treatment did not cause changes in their levels either. These observations suggest that the downward SMF can improve lipid metabolism and reduce the fatty liver in EtOH-fed mice.

### SMFs alleviate alcohol-induced inflammation and oxidative stress in the liver

**Fig. 3.**
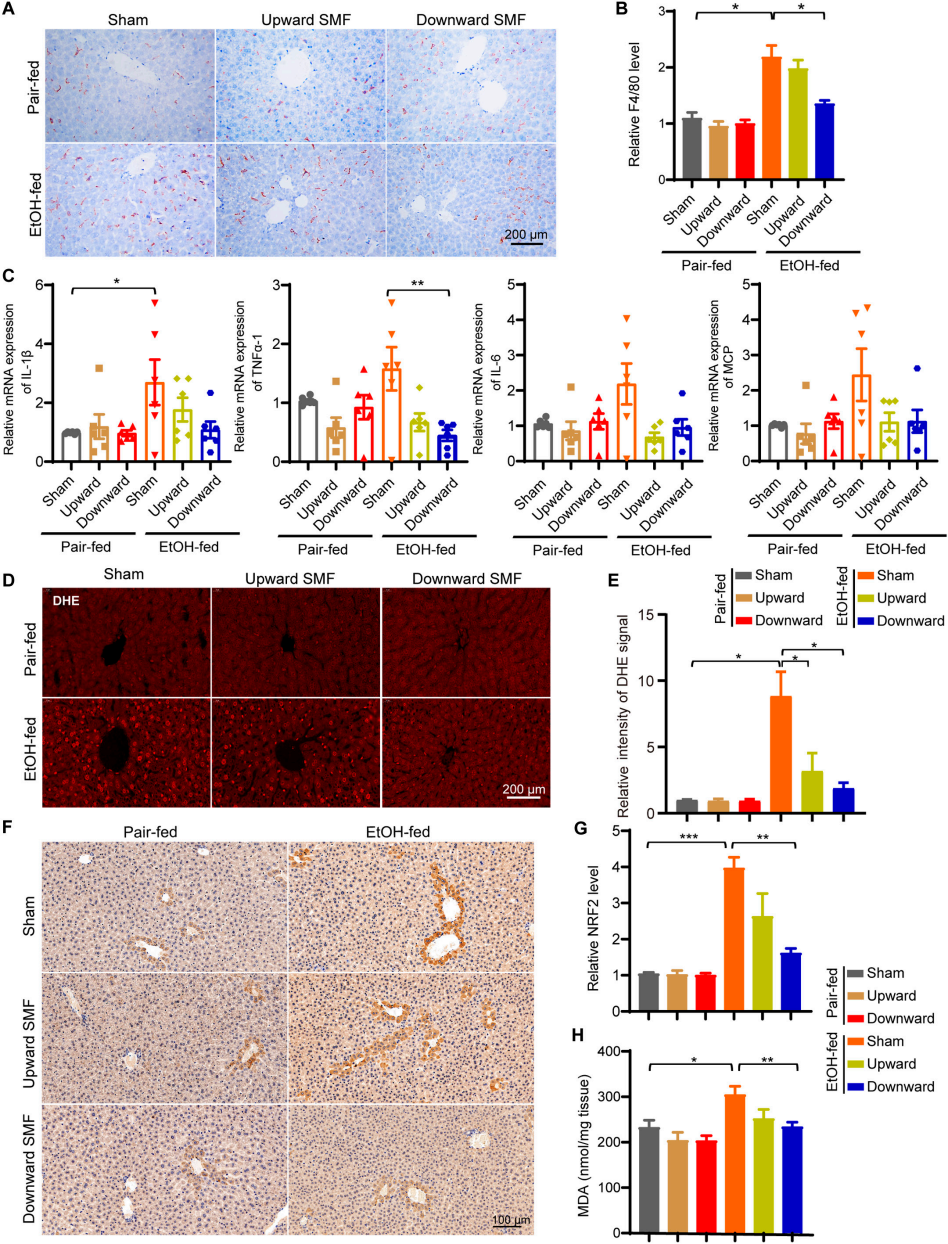
The downward SMF alleviates alcohol-induced inflammation and oxidative stress in liver. (A and B) Liver section immunohistochemical analysis of F4/80-positive cells and their quantification. (C) The mRNA expressions of proinflammation factors were detected by real-time fluorescence quantitative polymerase chain reaction, including IL-1β, IL-6, MCP, and TNFα-1. (D and E) Liver section immunofluorescent analysis of DHE-positive cells and their quantification. (F and G) Liver section immunohistochemical analysis of NRF2 and their quantification. (H) MDA in liver tissues was detected by the lipid peroxidation MDA assay kit. All values were expressed as means ± SEM. *n* = 3 to 5 per group. *P < 0.05, **P < 0.01, and ***P < 0.001 by Student's *t* test.

To examine liver inflammation, one of the main characters of ALD, we first used F4/80 immunohistochemistry staining to identify Kupffer cells, the liver-resident macrophages that play important roles in liver inflammatory responses. Consistent with the previous study [26], the Kupffer cells were increased in EtOH-fed mice (Fig. 3A and B). Moreover, SMFs significantly reduced the Kupffer cell numbers in EtOH-fed mice, especially the downward SMF (Fig. 3A and B). Furthermore, we used myeloperoxidase (MPO) antibody to identify neutrophils that were recruited to the liver in response to EtOH consumption. It was found that SMFs could also reduce the MPO-positive neutrophils in EtOH-fed mice, especially the downward SMF (Fig. S3A and B). We also examined the mRNA levels of proinflammatory cytokines in mouse liver, including interleukin-1β (IL-1β), IL-6, monocyte chemoattractant protein-1 (MCP-1), and tumor necrosis factor α-1 (TNFα-1). Significant cytokine mRNA level reductions were observed in EtOH-fed mice exposed to SMFs compared to sham group, especially the downward SMF (Fig. 3C). These results show that both SMFs can alleviate alcohol-induced liver inflammation, while the downward SMF had more obvious effects.

Since oxidative stress plays a central role in the development of ALD and magnetic fields have been shown to affect the ROS levels in multiple studies, we next measured the oxidative stress state in liver section. Using a dihydroethidium (DHE) fluorescent probe, we found that the alcohol-induced DHE fluorescence intensity elevation in mouse liver can be reduced by both SMFs, indicating lower oxidative stress, while the downward SMF had a more significant effect (Fig. 3D and E). We also examined the oxidative stress state using 2 other oxidative stress indicators, nuclear factor erythroid 2-related factor 2 (NRF2) and malondialdehyde (MDA). As expected, both NRF2 and MDA levels were increased in EtOH-fed mice (Fig. 3F to H). Consistent with the DHE staining, the upregulated NRF2 and MDA in EtOH-fed mice were both reduced by SMFs, especially by the downward SMF treatment (Fig. 3F to H). These results indicate that EtOH-induced oxidative stress can be reduced by SMFs, while the downward SMF treatment has a more significant effect.

### Upward SMF-induced ROS reduction was partially counterbalanced by the DNA changes in hepatocytes

**Fig. 4.**
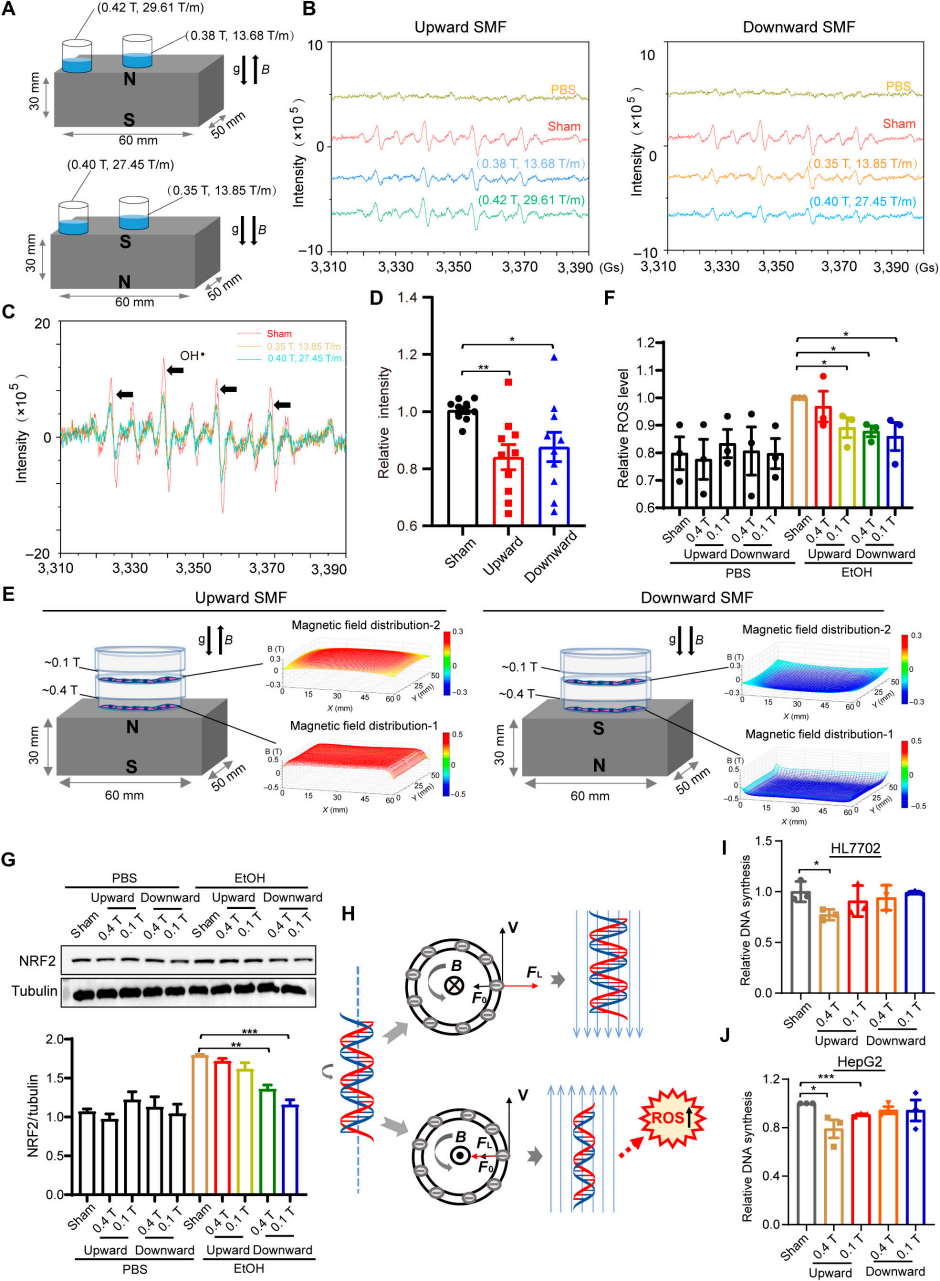
Free radical reduction was counterbalanced by the DNA perturbation of upward SMF in hepatocytes. (A) H_2_O_2_ solution (0.2 M) was placed at different positions on the surface of 2 magnets. (B and C) EPR experiments were used to detect and analyze the level of free radicals in H_2_O_2_ solution exposed to the upward SMFs. The black arrows indicate transitions correspond to the spin trap after scavenging hydroxyl radical. (D) EPR experiments were used to detect and analyze the level of free radicals in H_2_O_2_ solution exposed to the upward and downward SMFs. Quantifications of the OH· level were analyzed by Win EPR Acquisition software, *n* = 10. (E) Experimental setup and magnetic field distribution for cells exposed to upward and downward SMFs. (F) Flow cytometry measurement of cellular ROS of HL7702 cells exposed to 340 mM EtOH treated with sham, upward, or downward SMFs for 24 h. (G) Western blot analysis and quantifications of NRF2 in HL7702 cells exposed to EtOH and SMFs. (H) The pattern diagram of SMF and DNA. Cranked DNA motion and the magnetic Lorentz forces, (left) side view of DNA, and (right) top view of DNA cross section. For downward and upward SMFs, the Lorentz force (*F*_L_) of negatively charged DNA has different directions. (I and J) The synchronized HL7702 and HepG2 cells were exposed to SMFs for 8 h, and then DNA synthesis was detected by flow cytometer. All data were normalized, and values were expressed as means ± SEM, *n* = 3 to 5 per group. *P < 0.05; **P < 0.01; ***P < 0.001 by Student's *t*-test.

To further confirm the effect of SMF on oxidative stress and ROS, we set up a simple in vitro test to detect the changes of free radicals in H_2_O_2_ solution when exposed to different SMF conditions using electron paramagnetic resonance (EPR) (Fig. 4A to D). The 0.2 M H_2_O_2_ solution was placed at different positions on the magnet for 24 h, which have different magnetic field distributions (Fig. 4A). The EPR results showed that the free radical levels were decreased by the SMF treatment for both directions and both positions (Fig. 4B and D).

To investigate the effects of SMFs on the ROS and oxidative stress in hepatocyte, we exposed HL7702 cells to NdFeB magnets that were placed in the regular cell incubator (Fig. 4E). For cells on the magnet, the SMFs are in the range of 0.1 to 0.4 T. Furthermore, to analyze the effect of SMF on EtOH-induced cellular ROS elevation, we exposed HL7702 hepatocytes that were treated with 340 mM EtOH to SMFs for 24 h. The downward SMF significantly reduced EtOH-induced cellular ROS, while the upward SMFs were less effective (Fig. 4F). Since NRF2 is an oxidative stress marker, which may activate antioxidant enzyme to reduce intracellular ROS caused by alcohol-induced oxidative stress [27], we next examined the effect of SMF on cellular NRF2 level. Consistent with the liver tissue results, Western blot results and their quantifications confirmed that EtOH-increased cellular NRF2 protein level was significantly decreased by the SMFs, especially by the downward SMF (Fig. 4G). This is consistent with the ROS level reduction by the downward SMFs in H_2_O_2_ solution experiment (Fig. 4B to D). However, as shown in Fig. 4H, we have previously proposed that the upward SMF can inhibit cancer cell proliferation by inhibiting DNA synthesis by Lorentz forces exerted on the negatively charged DNA, which may elevate cellular ROS levels through increased DNA supercoil tightness. Here, we confirmed that the DNA synthesis in hepatocytes was also inhibited by the upward SMF, but not the downward SMF (Fig. 4I and J). This SMF-induced stress on DNA could also counterbalance the ROS reduction caused by SMF. Therefore, the downward SMF could have more marked effects in reducing cellular ROS and oxidative stress in hepatocytes.

### SMFs alleviate alcohol-induced cytotoxicity by reducing ROS levels

**Fig. 5.**
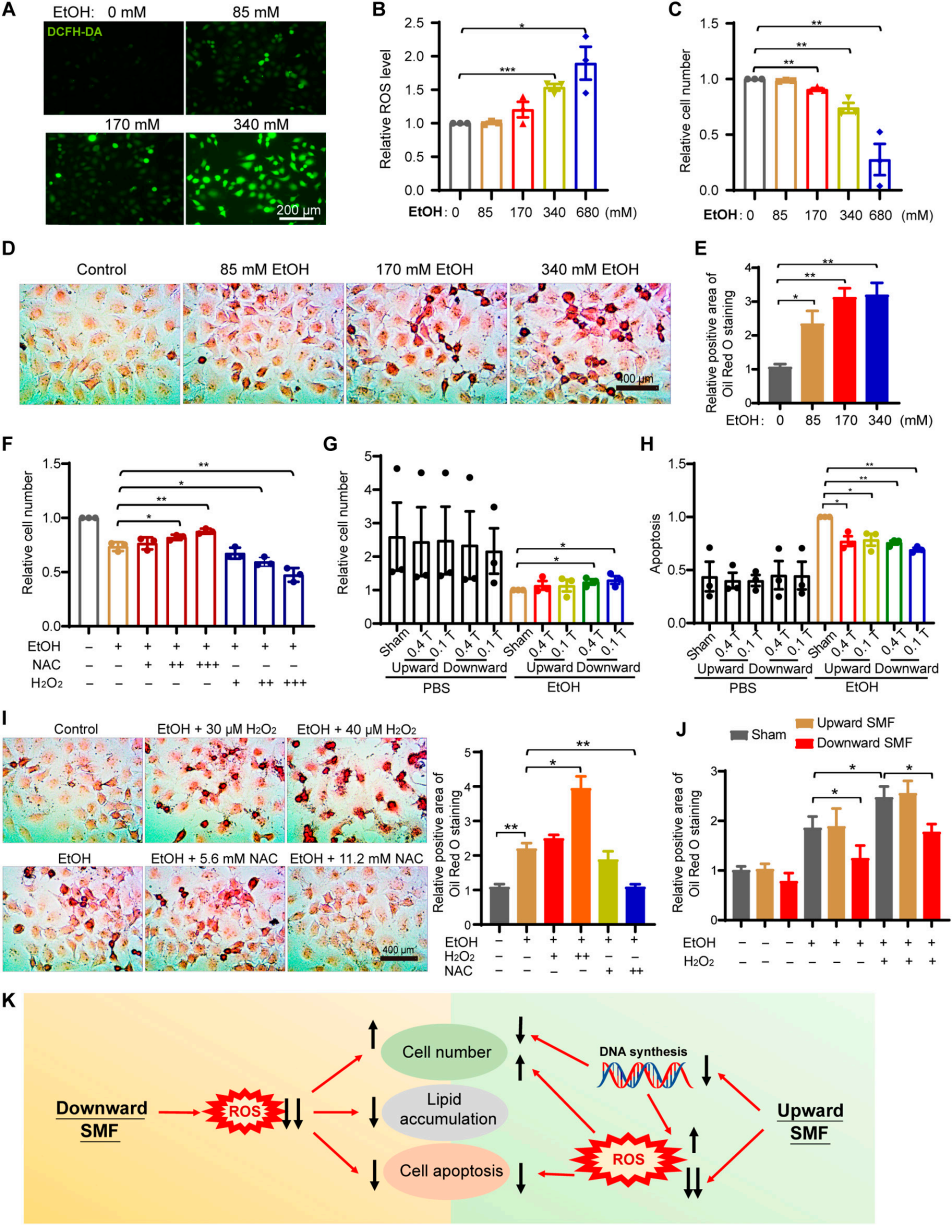
The downward SMF reverses the alcohol-induced hepatocytes injuries by reducing ROS level. (A) Representative fluorescent images of cellular ROS levels in 24-h EtOH-treated hepatocyte HL7702 cells using a DCFH-DA probe. (B) Flow cytometry measurement of ROS levels in 24-h EtOH-treated hepatocyte HL7702 cells using a DCFH-DA probe. (C) Cell number of HL7702 cells after 24-h EtOH treatment. (D and E) Representative images of Oil Red O staining and quantifications of HL7702 cells (4 × 10^4^ cells/ml) treated with different concentrations of EtOH for 24 h. (F) HL7702 cells (8 × 10^4^ cells/ml) exposed to 340 mM EtOH were treated with NAC (“+”, 2.8 mM; “++”, 5.6 mM; “+++”, 11.2 mM) or H_2_O_2_ (“+”, 20 μM; “++”, 30 μM; “+++”, 40 μM) for 24 h before their cell numbers were counted by flow cytometer. (G) Cell number quantification by flow cytometer of HL7702 cells (8 × 10^4^ cells/ml) exposed to 340 mM EtOH and sham, upward, or downward SMFs for 24 h. (H) Apoptosis analysis by flow cytometer using the annexin V-fluorescein isothiocyanate/propidium iodide stain of HL7702 cells exposed to 340 mM EtOH and sham, upward, or downward SMFs for 24 h. (I) Representative images of Oil Red O staining and quantifications of HL7702 cells (4 × 10^4^ cells/ml) treated with 340 mM EtOH and different concentrations of NAC or H_2_O_2_ for 24 h. (J) Quantifications of lipid accumulation in HL7702 cells (4 × 10^4^ cells/ml) treated with 340 mM EtOH and upward or downward SMFs for 24 h. (K) Mechanism illustration of the effect of SMF on the EtOH-induced cellular ROS, cell number, apoptosis, and lipid accumulation. The black arrows indicate levels increase or decrease. The number of arrows indicates the relative amount of changes. All data were normalized, and values were expressed as means ± SEM, *n*=3 per group. *P < 0.05; **P < 0.01; ***P < 0.001 by Student's *t*-test.

To further investigate the effects of SMF on alcohol-induced oxidative stress in hepatocytes, HL7702 cells were treated with different doses of alcohol for 24 h. We first confirmed that the ROS levels of the hepatocyte HL7702 cells were dose-dependently increased by EtOH treatment (*P* < 0.05) (Fig. 5A and B). Moreover, the cell numbers were dose-dependently decreased by EtOH treatment (*P* < 0.05), confirming the toxicity of EtOH treatment (Fig. 5C). To examine the effect of SMF on liver cell lipid accumulation, we used Oil Red O to stain HL7702 cells. Consistent with the mouse liver tissue results, the Oil Red O staining shows that alcohol could dose-dependently increase cellular lipid accumulation (*P* < 0.05) (Fig. 5E), confirming that ROS plays an important role in ALD.

Next, we examined the role of cellular ROS in EtOH-induced hepatocyte cell number reduction and lipid accumulation. Our results show that the ROS scavenger *N*-acetyl-l-cysteine (NAC) could reduce the toxicity of EtOH, while H_2_O_2_ further increased EtOH-induced HL7702 hepatocyte cell number reduction (Fig. 5F). Furthermore, the EtOH-induced hepatocyte cell number reduction (Fig. 5G) can be inhibited by SMFs, especially the downward SMF. The apoptosis can also be inhibited (Fig. 5H).

Meanwhile, we also found that the lipid accumulation could be significantly reversed by NAC or aggravated by H_2_O_2_, which demonstrated the correlation between ROS levels and lipid accumulation in hepatocytes (Fig. 5I). More importantly, the downward SMF obviously reduced cellular lipid accumulation induced by both alcohol and H_2_O_2_ (Fig. 5J). The effects of SMF on the EtOH-induced cellular ROS, cell number, apoptosis, and lipid accumulation are shown in Fig. 5K. This indicates that the downward SMF could reduce ROS levels and prevent alcohol or other oxidative-stress-induced fat deposition in hepatocyte. Therefore, our data show that SMFs could protect hepatocyte by reducing cellular ROS and oxidative stress.

### The RNA sequencing analysis reveals differential gene expression in EtOH-treated hepatocyte exposed to SMFs

**Fig. 6.**
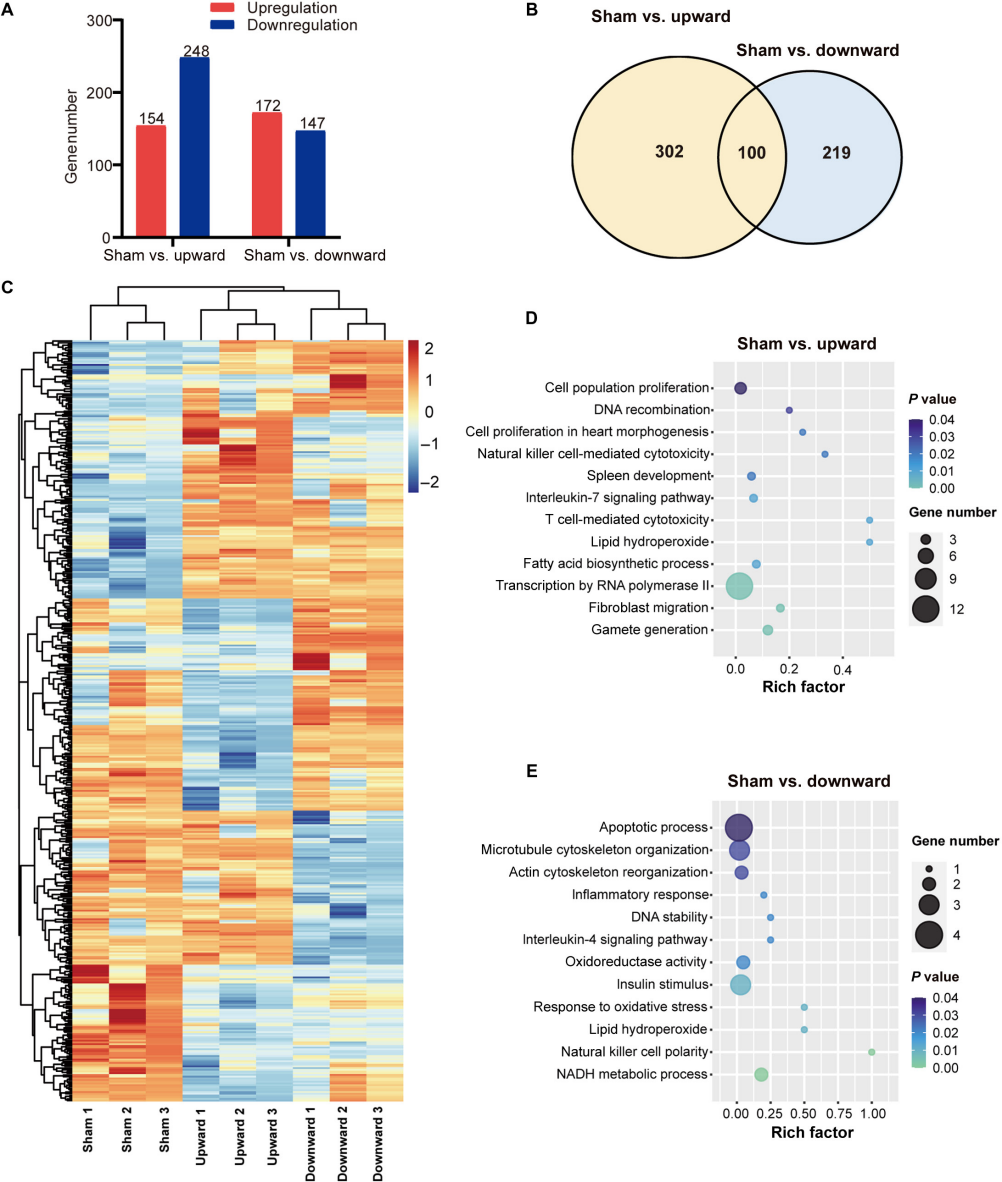
The RNA-seq analysis reveals differential expression of genome in EtOH-treated hepatocyte exposed to SMFs. (A) The differentially expressed genes from RNA-seq were screened by R package edgeR. (B) Venn diagram of the differential expression genes from 2 groups (sham versus upward and sham versus downward). (C) Heat map of the differentially expressed genes by RNA-seq. (D and E) GO term of the differentially expressed genes by RNA-seq after upward or downward SMF exposure. NADH, reduced form of nicotinamide adenine dinucleotide.

To further investigate the mechanism of SMFs on EtOH-treated hepatocyte and the differential effects of magnetic field direction, we performed RNA sequencing (RNA-seq) analysis on HL7702 cells after EtOH and/or magnetic field treatment. Our results show that when compared with the sham group, there were 154 upregulated and 248 downregulated genes when the cells were exposed to the upward SMF, while 172 upregulated and 147 downregulated genes when the cells were exposed to the downward SMF treatment (Fig. 6A). Among them, although there are 100 genes that were affected by both upward and downward magnetic fields (Table S1), the heat map of the differential expression shows that there are significant differences in their gene expression (Fig. 6C). Furthermore, the gene ontology (GO) term was used to evaluate the signaling pathways induced by the upward and downward SMFs. We found that the upward SMF was closely associated with cell proliferation and DNA recombination (Fig. 6D), while the downward SMF was closely associated with apoptosis and oxidative-stress-related pathways (Fig. 6E). In addition, we analyzed the 621 genes that were affected by the upward or downward SMFs by STRING database and found that they are enriched in cell cycle and mitotic regulations (Fig. S4), which confirms the effects of SMFs on cell proliferation.

### SMFs improve or deteriorate the survival of "heavy drinking" mice in a flux density and direction-dependent manner

**Fig. 7.**
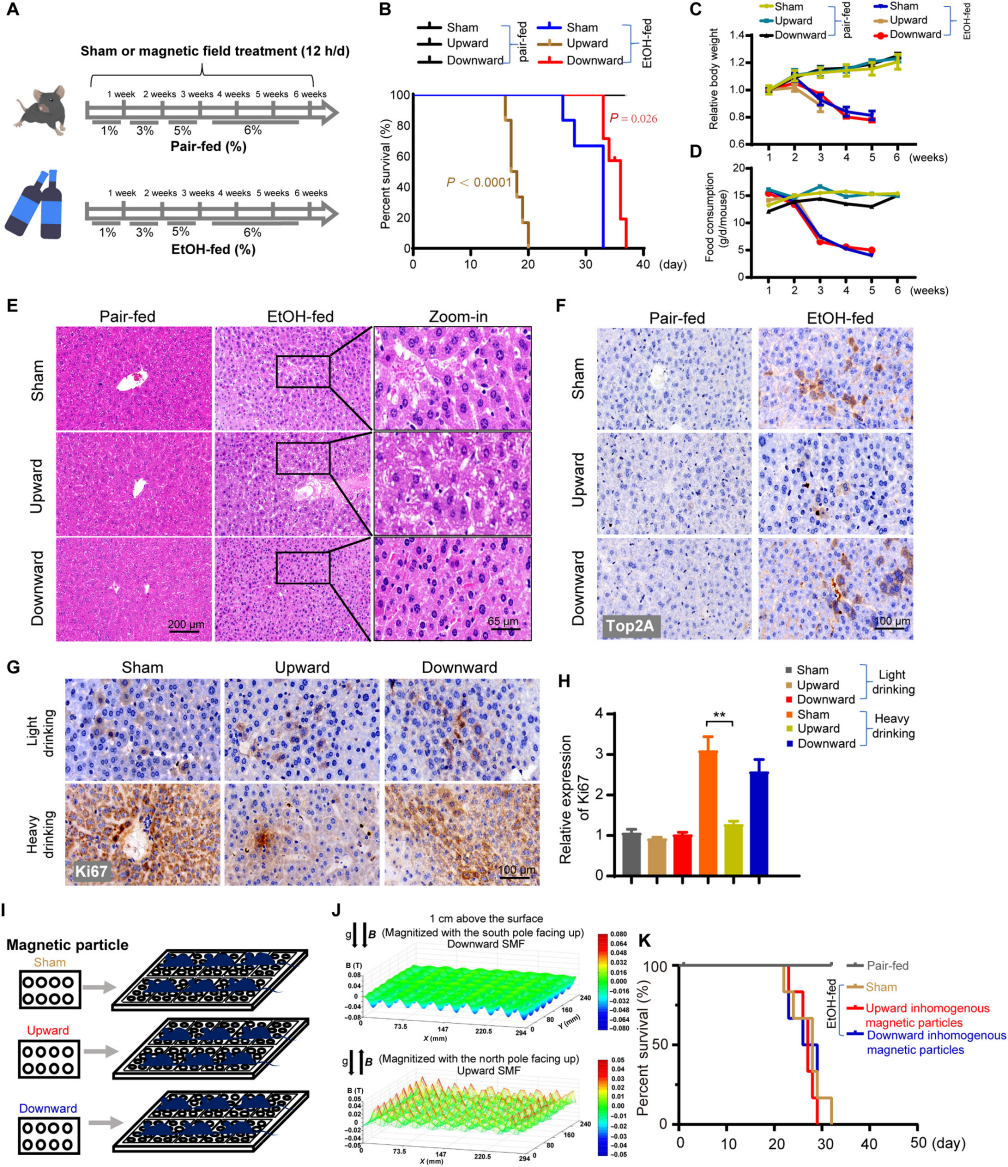
The magnetic field direction and flux density are both critical for the survival of "heavy drinking" mice. (A) The mice were exposed to a long-term EtOH-fed diet or pair-fed diet. (B) The survival rate of mice exposed to long-term diets. (C and D) Food consumption and body weight were recorded every week in sham, upward, and downward SMF groups. (E) After EtOH-fed mice died, paraffin-embedded liver sections were stained with H&E. Scale bars, 200 and 65 μm. (F) The expression of Top2A was detected in liver sections. Scale bar, 100 μm. (G and H) Liver sections were subjected to immunohistochemistry analysis using antibody Ki67. Quantified analysis of images by ImageJ software. Representative images are shown. (I) Unmagnetized or magnetized particles, with surface flux density of 0.5 T, were used to provide sham, upward, or downward inhomogeneous SMFs. (J) Magnetic flux distributions were scanned at 1 cm above the plates with a magnet analyzer. The whole mouse cage was place on the magnetic particles, and the SMF flux density at 1 cm above the plate is ~20 mT and that at 2 cm above the plate is ~7 mT, which are much weaker than the quasi-uniform SMFs provided by the magnetic plates. (K) The survival rate of mice exposed to magnet particles with "heavy drinking". **P < 0.01 by Student's *t*-test.

It is well known that excessive amount of alcohol consumption, heavy/binge drinking, can cause detrimental effects and lead to high death rate [28–31]. Our experiments above clearly show that moderate SMFs, especially the downward SMF, can reduce the oxidative stress and have obvious beneficial effects on alcohol-induced mouse liver lipid accumulation and toxicity. Here, we wanted to address whether SMFs can reduce the mortality of "heavy drinking" mice using an acute EtOH mouse model (Fig. 7A) [26]. Thirty-six mice were randomly divided into 6 groups with either EtOH-fed or pair-fed free access Lieber–DeCarli diet, with sham, upward, or downward SMF treatment. The lifespan of the "heavy drinking" mice in the sham group is 31.00 ± 2.88 d, while the pair-fed mice lived normally until the end of the experiment (Fig. 7B), which confirms the detrimental effects of excessive alcohol consumption.

Consistent with our previous results showing the beneficial effects of downward SMF on "light drinking" mice, the lifespan of "heavy drinking" mice was prolonged from 31.00 ± 2.88 to 34.83 ± 1.57 d (*P* < 0.05) (Fig. 7B). However, unexpectedly, the upward direction SMF exposure generated obviously detrimental effects on these alcohol-intoxicated mice (Fig. 7B), which is different from the subtle beneficial effects of the upward direction SMF exposure on "light drinking" mice. The lifespan is sharply reduced to 17.83 ± 1.34 d, which is 42.5% shorter than the sham control, after upward SMF exposure (Fig. 7B). All "heavy drinking" mice in the upward SMF group died before day 20. It should be mentioned that SMFs had no obvious effects on food consumption or body weight of these "heavy drinking" mice (Fig. 7C and D), which excluded the possibility that SMF-induced effects were due to increased EtOH intake. Moreover, to confirm this unexpected result, 2 researchers have repeated this experiment in a blinded way and confirmed that the upward SMF reduced the survival of "heavy drinking" mice (Fig. S5).

Next, we performed H&E staining for the heart, liver, spleen, lung, and kidney of the mice on day 37 (Fig. S6). Consistent with previous results in "light drinking" mice, the serious pathological damages induced by EtOH were alleviated by the downward SMF treatment (Fig. 7E). Interestingly, the immunohistochemistry results of topoisomerase 2A (Top2A) (Fig. 7F) and Ki67 (Fig. 7G) in the liver sections show that the "heavy drinking" mice have significantly increased Top2A and Ki67 levels. This indicates that there were active cell proliferation and liver regeneration after alcohol-induced liver cell damage. However, both Top2A and Ki67 were significantly downregulated by the upward SMF (Figs. 7F, 7G), indicating that the liver cell proliferation and liver regeneration were inhibited by the upward SMF. This is consistent with the findings of Yang et al. [32] that the upward SMF can inhibit DNA synthesis by Lorentz forces exerted on the negatively charged DNA. Gao et al. [33] also found that magnetic field could affect the self-assembly of DNA molecules by magnetic Lorentz force acting on the moving negative charges of DNA, which is closely associated with the flux density of magnetic field. Moreover, the data from RNA-seq analysis also showed that the upward SMF was closely associated with liver cell proliferation (Fig. 6D). We also compared the liver sections of "acute heavy drinking" and "chronic light drinking" mice for Ki67 immunohistochemistry to evaluate the liver regeneration (Fig. S7). It is obvious that the Ki67 staining level in "chronic light drinking" mice is much lower than the "acute heavy drinking" mice. Consequently, the Ki67 level was not significantly affected by the upward SMF in "light drinking" mice, but an obvious reduction was observed in "heavy drinking" mice (Fig. 7G and H). Consistently, the DNA synthesis at cellular level was also inhibited by the upward SMF (Fig. 4I and J).

Since the magnetic plates we used are relatively strong and not readily available for most people, we have also tested the effects of SMFs generated by arrays of magnetic particles that have 0.5 T surface intensity (Fig. 7I). Apparently, the field distribution of this type of magnetic fields is inhomogeneous (nonuniform) (Fig. 7J), which is drastically different from that of the quasi-uniform SMFs of the magnetic plates (Fig. 1A). Using the same experimental procedure, we found that these types of SMFs do not significantly affect the survival of heavy drinking mice (Fig. 7K). It should be mentioned that although the surface magnetic flux density of these magnetic particles is higher than the big chunk of magnetic plate (0.5 T versus 0.2 T), the flux density at 1 cm above the inhomogeneous magnetic particles is only 20 mT (max), while the flux density at 1 cm above the quasi-uniform magnetic plate is ~130 mT (average). At 2 cm above, where the back of the mice located, the flux density is ~70 mT (average) for the quasi-uniform magnetic plate but only 7 mT (max) for the inhomogeneous magnetic particles, indicating that the flux density of magnetic field is a key factor for the bioeffects on EtOH-fed mice. The SMFs below 20 mT has good biosafety, but the magnetic field above 70 mT should be vigilant and needs further research. The SMF gradient of the inhomogeneous magnetic particles at 1 cm above the plate is 0 to 1.2 T/m, which did not induce noticeable effects on mice.

## Discussion

**Fig. 8.**
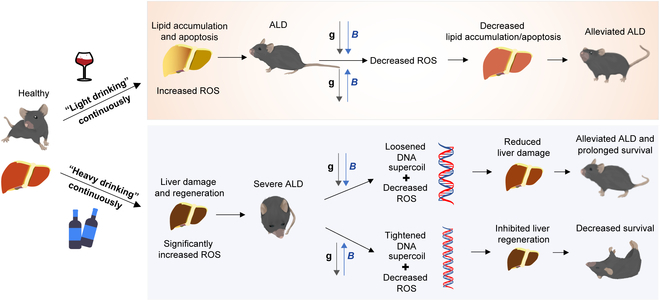
Mechanism of the upward and downward SMF treatment on "light drinking" versus "heavy drinking" mice.

We found that although 0.1 to 0.2 T quasi-uniform SMFs of both upward and downward directions could reduce the liver oxidative stress and inflammation, their overall effects on the alcohol-consuming mice are different. The exact effects depend on the alcohol consuming amount, magnetic field direction, and flux density, which differentially regulate cellular ROS and DNA synthesis, and therefore lead to differential cytotoxicity, lipid accumulation, and liver regeneration (Fig. 8).

Alcohol induces cellular ROS elevation and cell number reduction in a concentration-dependent way. Consequently, the liver is under very different conditions in "light drinking" versus "heavy drinking" mice. Our cellular experiments show that 85 mM EtOH did not have significant effect on cellular ROS or cell number, but 340 mM EtOH and above caused ROS elevation and cell number reduction very obviously. In mice experiments, it was also evident that the liver of the "heavy drinking" mice, but not the "light drinking" mice, underwent obvious cell proliferation. This is consistent with the previous finding that the expression of Ki67 in the liver was upregulated by alcohol [34]. This is likely due to liver regeneration after heavy alcohol consumption. We found that SMF-induced DNA synthesis and cell proliferation inhibition are key reasons that impaired liver regeneration after "heavy drinking". We have previously shown that the upward SMFs could affect the DNA rotation and supercoil tightness by aligning DNA chain and exerting Lorentz forces on negatively charged DNA, which inhibited DNA synthesis, but not downward SMF [32,35]. Panczyk and Camp studied the effect of the Lorentz force on DNA structure by 0.5 T SMF and found that the mean DNA radius was slightly affected by SMF in nanoseconds [36]. Since we have put the mice on magnetic plate for weeks, it is a long-term effect of the competition of the magnetic forces with that of thermal fluctuations. This effect will lead to inhibited cell proliferation, tumor growth inhibition, and liver cell regeneration. Our results showed that the upward SMF decreased the expression of Ki67 and Top2A in EtOH-fed mice. We speculated that the hepatocyte regeneration was inhibited by the upward SMF in EtOH-fed mice. Therefore, the EtOH-fed mice had a low survival rate when exposed to the upward SMF.

The alleviated oxidative stress and inflammation, as well as reduced lipid levels in serum and improved ALD by SMFs in our study, are likely the consequences of reducing ROS. Alcohol and its metabolites have severe hepatotoxicity and can cause oxidative stress and inflammation, which significantly increased serum ALT, AST, and TG in mice. It is known that the oxidative stress is mediated by cellular ROS in alcohol associated liver disease [37]. We think that the ROS levels are affected by SMFs through a combination of radical pair mechanism and DNA stress. Although SMFs affecting free radicals in biological systems have been reported in many studies, the exact effects or mechanism still remain elusive. The magnetic field effects due to the radical pair mechanism are insensitive to the inversion of the magnetic field direction. Therefore, it is not surprising that our in vitro EPR experiments did not reveal differences between the upward versus downward SMFs on free radical generation from H_2_O_2_. However, although the moderate SMFs of both upward and downward directions can reduce the liver oxidative stress and inflammation in vivo, the downward SMF had much more marked effects. This is probably due to the tightened supercoil of the DNA caused by upward SMF, which induces extra stress to the cells. The oxidative metabolism of alcohol generates an obvious increase in ROS [38], and ROS-induced oxidative stress is a major factor that causes liver injury [37]. It is known that change of the radical-pair recombination will affect the generation of free radicals [39,40], which can be affected by SMFs in a field-intensity-dependent way [19]. However, the exact effects of SMFs on ROS levels in cells are cell type dependent [21]. In fact, there are many studies that have found the influence of magnetic fields on ROS [18], but people still do not have an unambiguous conclusion about the exact consequences on ROS level or the mechanisms that caused the differential effects. Our in vitro EPR studies of H_2_O_2_ solution showed that the free radical level was significantly reduced by moderate SMFs. However, it is still unclear about how the magnetic field effects on unpaired electron spin state connect to free radical generation, neutralization, and redox regulation of the proteome in a reliable and predictable way and how to improve or worsen of specific clinical conditions in a reliable and predictable way.

The novelty of our work is the harmful effects of moderate SMFs on "heavy drinking" mice. Most studies of SMFs have reported various beneficial effects, indicating their potentials in magnetotherapy. For example, we recently found that the exactly same sets of magnetic plates in this study not only can prolong the lifespan and health span of normally aged mice [41] but also can alleviate the kidney injury induced by cisplatin [42]. The guideline provided by World Health Organization (WHO) and International Commission on Non-Ionizing Radiation Protection (ICNIRP) set the safety threshold for the public exposure of SMF to 400 mT based on available research evidences [43]. However, most previous studies were performed on healthy subjects or animal models of a few mild pathological conditions. Here, we investigated the effects of SMFs on alcohol "light drinking" versus "heavy drinking" mice, which has never been studied before. It should be mentioned that the ICNIRP updates their guidelines for radiofrequency magnetic fields related to the cell phone, microwave, etc., from 100 kHz to 300 GHz about every 2 years, because there are a large number of relative studies. In contrast, since SMFs are considered much safer and there are very limited safety studies, the guideline for SMFs was lastly published in 2009 and has not been updated since then. Besides this study, we also recently found that gradient high SMF (>10 T/m) could generate harmful effects on diabetic mice, especially for severe type 1 diabetic mice [44]. Since SMFs have the advantages of high penetration and no attenuation crosses the living organisms, they usually generate similar magnetic forces in small- or large-sized organisms because the molecules and cells are similar in size. Therefore, our study will be helpful to alert the field and calls for more systematic studies for severe pathological conditions under 400 mT and helpful for WHO and ICNIRP to modify their standard and to alarm the use of magnetic field on human bodies in applications including magnetic resonance imaging in hospitals and magnetotherapy.

In summary, our study revealed that a 0.1 to 0.2 T quasi-uniform SMF with a downward direction could alleviate ALD through reduced ROS, oxidative stress, and inflammation. Unexpectedly, the upward 0.1 to 0.2 T quasi-uniform SMF setting in our study may be a taboo for heavy drinkers, which might be a potential precaution to some open magnetic resonance imagings that usually have upright magnetic field of ~0.5 T. On one hand, our study and multiple studies in the literature have illustrated the potential to develop magnetic field as a physical countermeasure. However, on the other hand, and more importantly, our study unraveled some potential detrimental effects of these magnetic fields for severe pathological conditions, which will help WHO and ICNIRP to modify their safety guidelines for magnetic field exposure.

## Materials and Methods

### Mouse model

Eight-week-old male BALB/c mice purchased from GemPharmatech Co. Ltd. (Nanjing, China) were used in this study. All mice were randomly divided into EtOH-fed groups (sham, upward, and downward SMFs; EtOH diet) and pair-fed groups (sham, upward, and downward SMFs; maltodextrin diet). Lieber–DeCarli diets (products L10015 and L10016) were prepared fresh daily and fed ad libitum to mice using a short-term (chronic EtOH-fed mouse model with "light drinking") and long-term (acute EtOH-fed mouse model with "heavy drinking") EtOH feeding protocol of ALD according to the previous report [26]. The formulas of diets were formulated by Research Diets. The mice were freely exposed to sham, upward, and downward SMFs in acrylic cages for 3 weeks (chronic model) or 6 weeks (acute model) in a pathogen-free facility under conditions of controlled temperature (22 to 23 °C), humidity (40% to 60%), and illumination (12 h light/dark cycle). In the acute EtOH-fed mouse model, the diet containing EtOH was designed as 1% EtOH for 1 week, 3% EtOH for 1 week, 5% EtOH for 1 week, and 6% EtOH for 3 weeks. In the chronic EtOH-fed mouse model, the diet containing EtOH was designed as 1%, 2%, and 3% EtOH for 1 week, respectively. The diet consumption and body weight were recorded during the whole experiment. After the mice were executed, serum and organs were collected and stored at −80 °C. All animal experiments were conducted according to the National Institutes of Health Guide for the Care and Use of Laboratory Animals (NIH publication no. 8023, revised 1978) and approved by the ethical and humane committee of Anhui Medical University (license # LLSC 20211043) (Hefei, China).

### Magnetic field setup

The upward and downward quasi-uniform SMFs were provided by permanent magnet plates (length × width × height = 250 mm ×160 mm × 45 mm). Each magnetic plate was composed of 12 neodymium magnet cubes (length × width × height = 60 mm × 50 mm × 30 mm) that were purchased from Sans (Nanjing, China). The surface magnetic flux densities of the magnet cubes are 0.22 T. These magnet plates can produce a relatively uniform SMF at each horizontal plane (1 cm above the plate, ~130 mT; 2 cm above the plate, ~70 mT).

The upward and downward inhomogeneous SMFs were provided by fixing arrays of neodymium magnet particles (diameter × height = 16 mm × 7 mm) on a plate. The surface magnetic flux densities of these magnet particles are 0.5 T. These magnet particles can produce inhomogeneous SMFs at each horizontal plane (1 cm above the plate, ~20 mT max; 2 cm above the plate, ~7 mT max).

Groups of experimental mice were continuously exposed to SMFs for 12 h/d during the light and dark cycle. To measure the distributions of magnetic field at the mouse location, a magnet analyzer (FE-2100RD, Forever Elegance, China) was used to scan the planes at different levels above the magnets. In addition, cultured cells were also exposed to neodymium magnets (N38, length × width × height = 60 mm × 50 mm × 30 mm) with the surface magnetic flux density of ~0.4 T.

### H&E staining

At the end of the experiment, all mice were dissected to collect organs including the heart, liver, spleen, lung, and kidney. Then, organs were fixed and processed with formalin to obtain 5 μm-thick sections and stained with H&E. Five random areas were examined in each section.

### TUNEL assay

TUNEL detection kit (C1098, Beyotime, China) was used to detect apoptotic cells in liver tissue. 3,3′-diaminobenzidine reagent was stained and incubated at room temperature for 5 to 30 min. Liver sections of TUNEL staining were quantitatively analyzed by counting TUNEL-positive cells from at least 5 microscopic areas.

### DHE staining

DHE dissolved in dimethyl sulfoxide was used to detect ROS in mouse liver. The frozen sections of the liver were treated with phosphate-buffered saline (PBS) for 10 min and tissue autofluorescence quencher, then 100 μl of staining working solution (DHE dilution, 1:1,000) was added by drop, and the samples were incubated at 37 °C for 60 min in the dark. Finally, the sections were stained with 4′,6-diamidino-2-phenylindole and antifluorescence quencher seal.

### Oil Red O staining

Oil Red O staining solution (R23104, Baso, China) was used to detect lipid accumulation in liver sections and human hepatocyte HL7702 cells (American Type Culture Collection). HL7702 cells were seeded in 35 mm cultured dishes, treated with EtOH (0, 85, 170, and 340 mM) or magnetic fields (sham, upward, or downward SMFs) for 24 h. Oil Red O was mixed with double-distilled H_2_O at the ratio of 3:2 and left at room temperature for 10 min. Oil Red O staining solution and 70% propylene glycol were added with agitation for 5 to 10 min, followed by washing in running water for 30 s. Hematoxylin solution was used for staining for 2 min and then washed off with running water for 5 min. At last, stained sections and cells were examined by microscopy.

### Blood chemistry and tissue MDA tests

The blood samples were collected in 1.5 ml centrifuge tube at 4 °C for 15 min at 3,500*g* to collect serum, which were analyzed by an automated biochemical analyzer (HITACHI 7020, Japan). Lipid peroxidation MDA assay kit (S0131S, Beyotime, China) were used to detect the MDA concentration in liver tissues according to the manufacturer's instructions.

### Immunohistochemistry

Liver section immunohistochemistry (NeoBioscience, ENS004120) was performed using the immunohistochemistry antibodies for NRF2 (abs130481, Absin), MPO [79623S, Cell Signaling Technology (CST)], Ki67 (27309-1-AP, Proteintech), Top2A (202331-1-AP, Proteintech), and F4/80 (70076S, CST). All the steps were performed according to the manufacturer's instructions. Quantitation of staining was evaluated by ImageJ analysis of at least 5 microscopic areas.

### Cell number counting and apoptosis analysis by flow cytometry

Human hepatocyte HL7702 cells were exposed to alcohol and magnetic fields before they were collected in 1.5 ml centrifuge tube by 0.25% trypsin digestion. Cell number was counted by flow cytometry. In addition, cultured cells were exposed to alcohol and SMFs for 24 h. Annexin V-fluorescein isothiocyanate apoptosis detection kit (0076884, BD Biosciences, USA) was used to analyze hepatocyte apoptosis according to the manufacturer's protocol. Cell apoptosis was detected by flow cytometry.

### Western blot analysis

The protein isolations were collected with protease inhibitors from liver tissues and cultured cells, and Western blotting was performed as previously described. The proteins transferred to polyvinylidene fluoride membranes were incubated with primary antibodies NRF2 (12721S, CST), BCL2 (26295S, CST), cleaved caspase3 (9664S, CST), caspase3 (9662S, CST), anti-tubulin (2128S, CST) and anti-glyceraldehyde-3-phosphate dehydrogenase (5174S, CST). ImageJ software was used to quantitatively analyze the expression of proteins.

### Reverse transcription and quantitative polymerase chain reaction

Mice exposed to the short-term alcoholic diet were executed to collect liver tissues. In addition, liver tissues were polished on ice and were used to extract RNA by an RNeasy animal RNA isolation kit with spin column (R0027, Beyotime). Novoscript plus all-in-one 1st strand cDNA synthesis supermix (E047-01A, Novoprotein) was used to RNA reverse transcription, and Novostart SYBR qPCR supermix plus (E096-01A, Novoprotein) were used to amplify target gene under the action of specific primers. The detailed primers sequences were shown in Table S2.

### Cellular ROS detection

HL7702 cells (8 ×10^4^ cells/ml) were seeded in 35-mm culture dishes and supplied with complete medium. After attachment, the medium was supplemented with different concentrations of EtOH (0, 85, 170, and 340 mM) and/or exposed to sham, upward, or downward SMFs for 24 h. ROS detection kit (D6883, Sigma-Aldrich) containing 2′,7′-dichlorodihydrofluorescein diacetate (DCFH-DA) was used to detect cellular ROS. DCFH-DA can freely penetrate the cell membrane and is hydrolyzed by esterase in the cell to produce DCFH that cannot pass through the cell membrane, while ROS can oxidize nonfluorescent DCFH to produce fluorescent DCF. Cultured cells were incubated with 10 μM DCFH-DA at 37 °C for 30 min. Green fluorescence can be observed by a fluorescence microscope, and the intensity of green fluorescence is directly proportional to the level of cellular ROS. Meanwhile, flow cytometry and confocal fluorescence microscope were used to evaluate the intensity of fluorescent DCF.

### RNA-seq analysis

HL7702 cells were treated with 340 mM EtOH and exposed to ~0.4 T upward and downward SMFs in the permanent magnet plate or the sham for 24 h before being collected and frozen at −80 °C with RNAiso Plus (Takara, Japan). Total RNA was extracted, and a genome-wide transcriptomics analysis was conducted by LC-Bio Technology Co. Ltd. (Hangzhou, China). After the final transcriptome was generated, StringTie and ballgown (http://www.bioconductor.org/packages/release/bioc/html/ballgown.html) were used to estimate the expression levels of all transcripts by calculating FPKM {FPKM = [total_exon fragments/mapped_reads (millions) × exon_length (kB)]}, (command line: ~stringtie-e-B-p 4-G merged.gtf-osamples.gtf samples.bam). The differentially expressed mRNAs were selected with a fold change of >1.5 or <0.65 and *P* < 0.05 by R package edgeR (https://bioconductor.org/packages/release/bioc/html/edgeR.html) or DESeq2 (http://www.bioconductor.org/packages/release/bioc/html/DESeq2.html). GO term of the genes with differential expression was performed with LC-Bio Technology Co. Ltd. (https://www.omicstudio.cn/tool). Experiments were performed and analyzed in a blinded way.

### EPR experiment

H_2_O_2_ solution (500 μl, 0.2 M) was made by mixing 10 μl of H_2_O_2_ (10 M, Analytical Reagent ≥ 30%) and 490 μl of PBS buffer (137 mM NaCl, 2.7 mM KCl, 10 mM Na_2_HPO_4_, and 1.8 mM KH_2_PO_4_) and was then exposed to the SMFs for 24 h. 5,5-dimethyl-1-pyrroline *N*-oxide (92688, Sigma-Aldrich) was added to the solution for 5 min before EPR experiments. Then, equal amount of solution was immediately added to the test capillary. The free radical levels from the solutions loaded into a capillary from each condition were tested by the EPR device (Bruker EMX plus 10/12, Switzerland), Q factor of ~1,200, equipped with Oxford ESR910 Liquid Helium cryostat. The EPR experiments were repeated 10 times in a blinded way.

### DNA synthesis assay

HL7702 and HepG2 cells (8 ×10^4^ cells/ml) were seeded in 35 mm dish, synchronized with 2.5 mM thymidine for 16 h, and then treated with 10 μM 5-bromo-2′-deoxyuridine (Sangon, E607203) for 8 h when they were exposed to SMF or sham at indicated time point. Then, all cells were fixed by 70 % EtOH for 12 h, washed by PBS, resuspended by 2 M HCl, incubated on the rotator for 30 min at room temperature, centrifuged, and resuspended by 0.1 M Na_2_B_4_O_7_ (pH 8.5) at room temperature for 10 min before they were washed by PBS. Finally, the cells were incubated with anti-5-bromo-2′-deoxyuridine antibody for 2.5 h and the secondary Alexa Fluor 488-conjugated antibody for 1.5 h. All cells were analyzed by flow cytometry.

### Statistical analysis

All statistical analysis was performed using GraphPad Prism version 8. Dates from the experiments were shown as the means ± SEM. The *P* values were calculated using the 2-tailed paired or unpaired Student's *t* test for comparisons. *P* < 0.05 was considered statistically significant.

To reduce the potential experimenter bias and unreproducible results, we have always performed our experiments in a blinded way or have repeated the experiments by at least 2 independent researchers. Their results were pooled together for statistical analysis, or representative images/results were selected for the figures.

## Data Availability

All data needed to evaluate the conclusions in the paper are present in the paper and/or the Supplementary Materials. Additional data related to this paper may be requested from the authors.

## Supplementary Materials

Supplementary 1 Physiological index detection Figs. S1 to S7 Tables S1 and S2
