# Supplementary material for: Magnetic Fields Affect Alcoholic Liver Disease by Liver Cell Oxidative Stress and Proliferation Regulation
Source: Research (Wash D C). 2023 Mar 30;6:0097. doi: 10.34133/research.0097 (PMC10063227; doi:10.34133/research.0097)
Supplement: Supplementary 1 — Physiological index detection Figs. S1 to S7 Tables S1 and S2 [file research.0097.f1.docx]

Supplementary Materials

**Physiological index detection**

The physiological condition of mice was monitored by the small animal vital signs monitor (STARR Life Science, USA). The signal sensor was placed on the necks of the mice, their hair can affect signal reception. Therefore, BALB/c mice with white hair were selected to perform the experiment. Mice were placed in the cage and monitored for 6 min. The breath rate, pulse distention, heart rate and arterial O_2_ were calculated using Mouse-ox plus software (STARR Life Science, USA).

**Supplementary Figures**


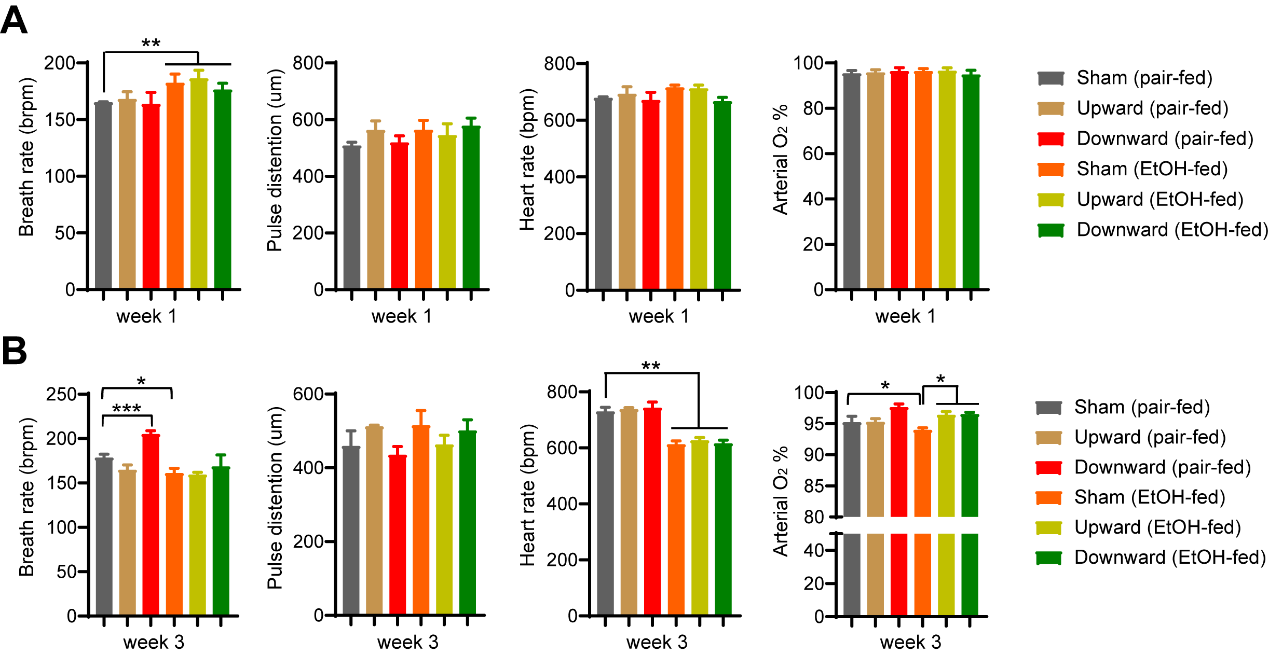


**Fig. S1. The effects of magnetic field on short-term EtOH-fed mice with light drinking. (A)** Heart rate, breath rate, pulse distention and arterial O_2_ were monitored by a small animal vital sign monitor at the first week. **(B)** Heart rate, breath rate, pulse distention and arterial O_2_ were monitored at the third week. Values represent means ± SEM, *n*=6 per group. *, *P*<0.05; **, *P*<0.01; ***, *P*<0.001 by Student’s *t*-test.


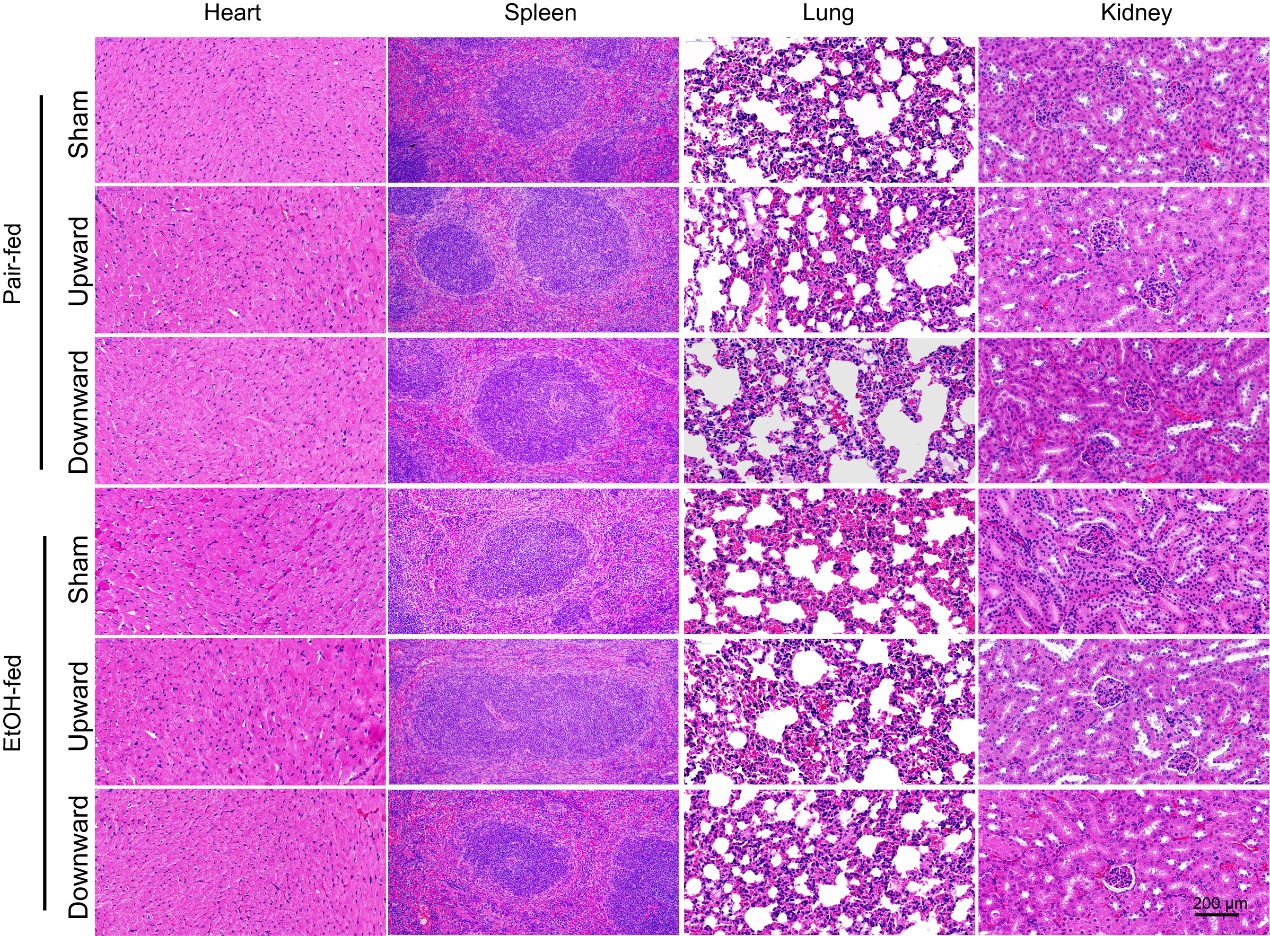


**Fig. S2. The effects of magnetic field on short-term EtOH-fed mice with light drinking.** Heart, spleen, lung and kidney were analyzed by H&E staining in mice exposed to long-term alcoholic diet. Representative images are shown. Scale bar: 200 µm.


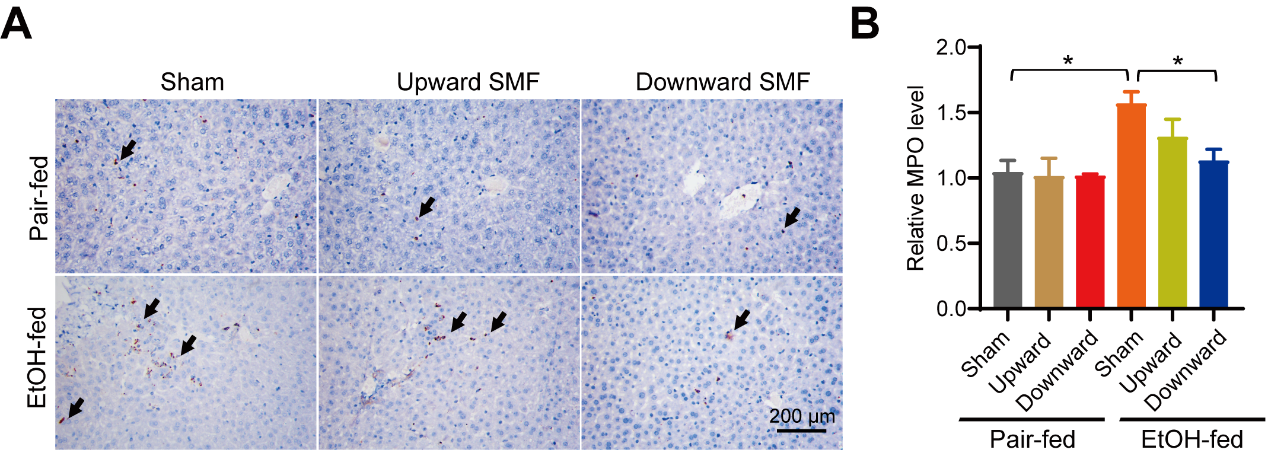


**Fig. S3.**  **MPO analysis of liver from the light drinking mice.** **(A, B)** Mice liver section immunohistochemical analysis of MPO positive cells and their quantification. *, *P*<0.05 by Student’s *t*-test.


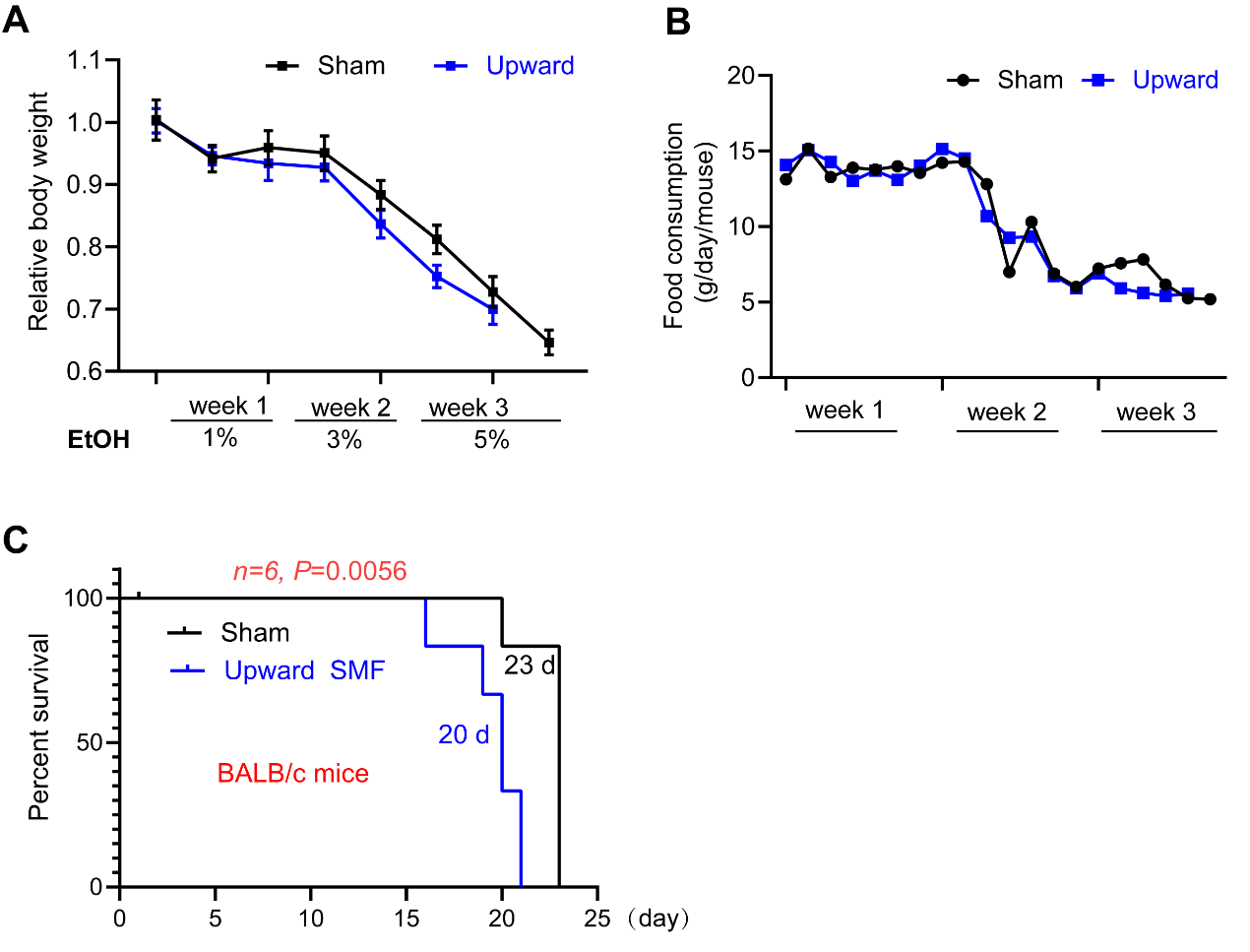


**Fig. S4.** **Repeated experiment confirming that the upward SMF decreased the survival in heavy drinking mice.** The experiments were set up identically to Figures 7A-7C to confirm the observed results. **(A-B)** The mice were exposed to a long-term EtOH-fed diet and upward SMF for 3-4 weeks. Body weight and food consumption were recorded every week in sham and upward groups. **(C)** The survival rate of mice exposed to long-term diets with heavy drinking.


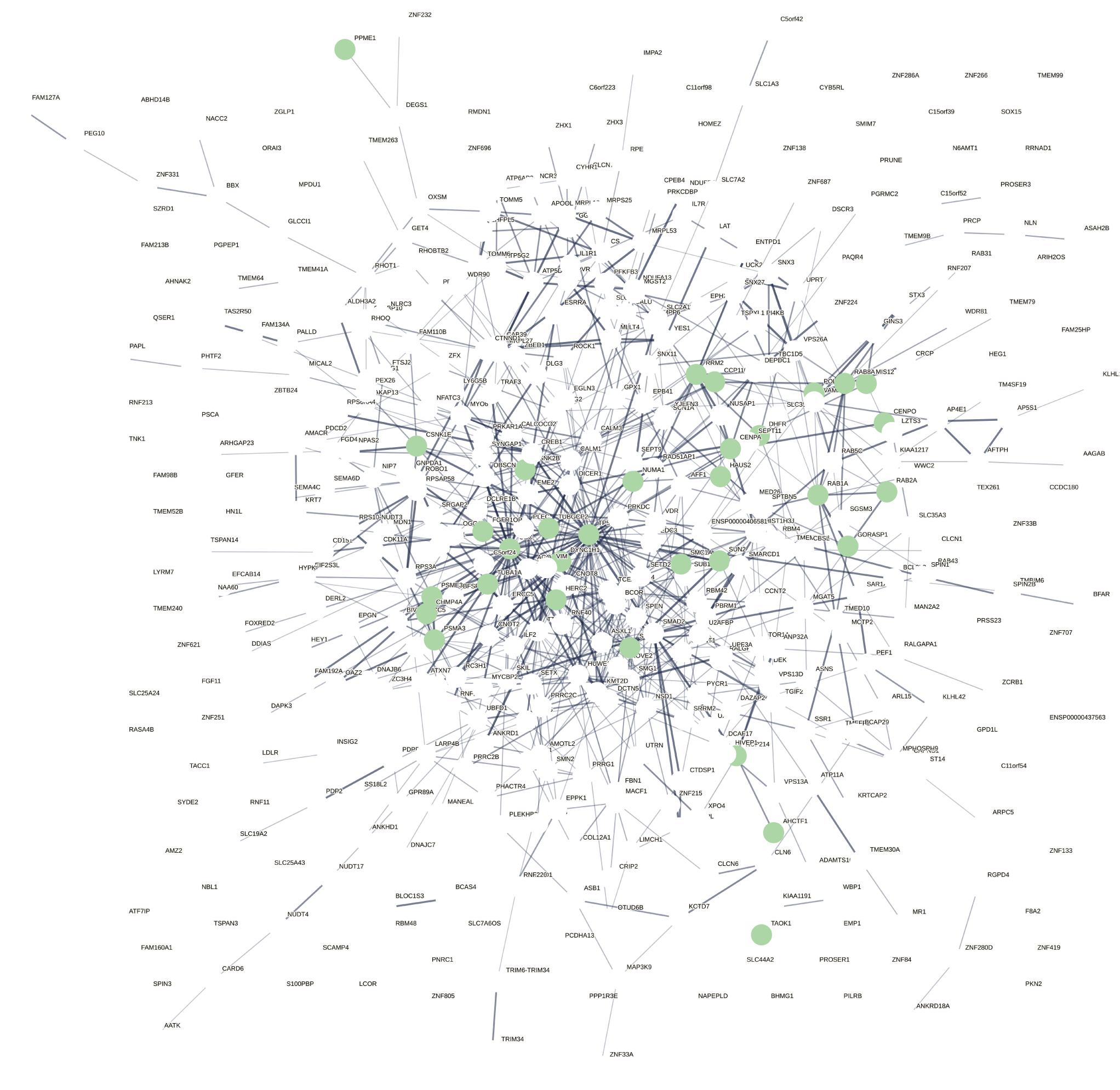


**Fig. S5. 621 genes that were differentially expressed in either upward or downward SMFs were analyzed by STRING database to evaluate protein interaction and signaling pathways.**


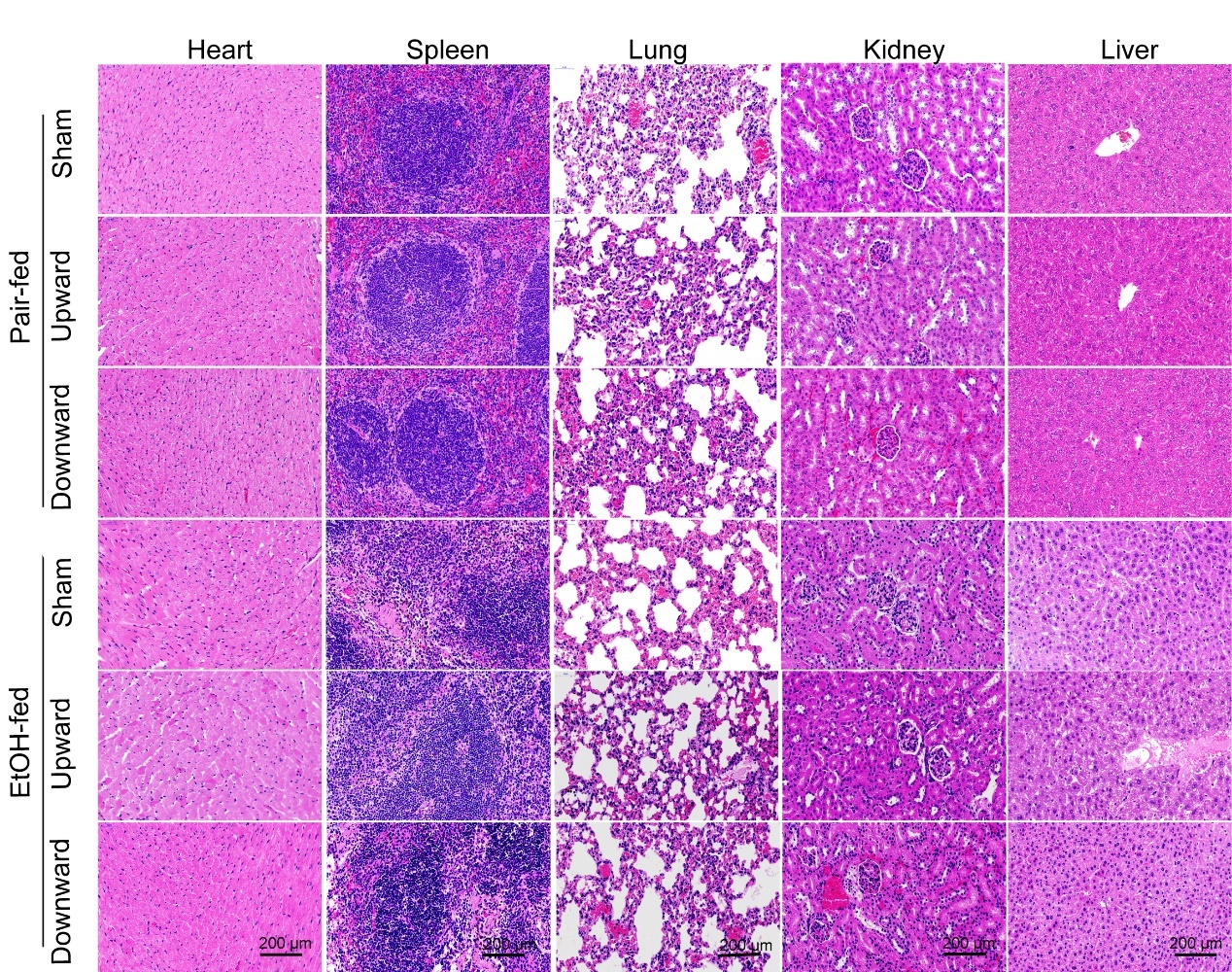


**Fig. S6. The effects of magnetic field on long-term EtOH-fed mice with heavy drinking.** Heart, spleen, lung and kidney were analyzed by H&E staining in mice exposed to long-term alcoholic diet. Representative images are shown. Scale bar: 200 µm.


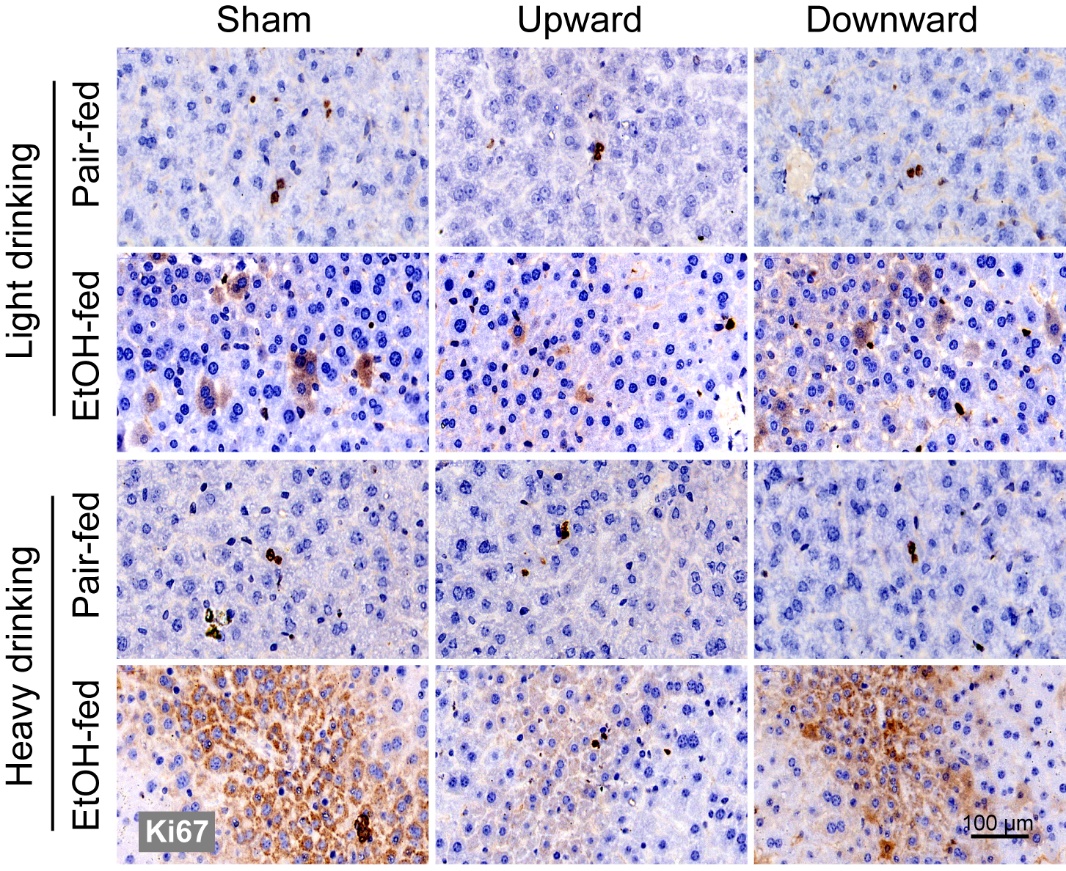


**Fig. S7.**  **The upward SMF reduced the expressions of Ki67 in EtOH-fed mice.** Liver sections were subjected to immunohistochemistry analysis by using Ki67 antibody. Representative images are shown. Scale bar: 100 µm.

**Supplementary Tables**

**Table 1. 100 commonly differential expression genes by upward and downward SMFs**

| Gene name | | | | |
| --- | --- | --- | --- | --- |
| AP003071 | ZNF791 | IBA57 | PEX2 | CNOT9 |
| LINC00205 | SAMD4A | LRRC8D | CDK18 | CDC25B |
| AC093525 | RBPJ | MPZL1 | NAV2 | G3BP2 |
| PCDHA11 | FAM111A-DT | AC021087 | LRRC37A4P | CSNK1G3 |
| CU633904 | APH1B | MKLN1 | RIF1 | RPL14 |
| AC244197 | AC010323 | ZBED6 | ITPR2 | ARF4 |
| RFX7 | HOXC-AS2 | AC055811 | NPR3 | MYO5A |
| FER | MIR503HG | MUC16 | TRIO | PRELID1 |
| PPAN-P2RY11 | PRR5L | MOGS | NBDY | CHMP1A |
| ZDBF2 | ABHD10 | ARNTL2 | TCF3 | BIRC2 |
| PRICKLE4 | KCTD20 | ARL8B | SNRPD2 | ADD3 |
| AC087632 | GPX3 | IL4R | MXI1 | GORASP2 |
| ZNF260 | RBM15 | ZSCAN31 | FOXP2 | DHCR24 |
| RUNX2 | NRAV | MTMR3 | NDUFA7 | FP236383 |
| AL365205 | SNORD36A | GABARAPL1 | IL10RB | UCK2 |
| POMK | SNHG20 | FAM219B | HRH1 | FP671120 |
| UTP14C | GEN1 | GRPEL2 | EHBP1L1 | ACP1 |
| FTH1P10 | PLP1 | LINC01116 | AHSA2P | ETNK1 |
| AP001931 | SLC22A23 | RNASEH2C | RTEL1-TNFRSF6B | TYW5 |
| AC011448 | TWNK | FAM217B | ATF3 | AC015813 |

**Table 2. Sequence based reagents**

| Name | Sequence | Supplier |
| --- | --- | --- |
| M-TNFα | F-CGTCAGCCGATTTGCTATCT  R-CGGACTCCGCAAAGTCTAAG | Sangon, China |
| M-IL-1β | F-GCAACTGTTCCTGAACTCAACT  R-ATCTTTTGGGGTCCGTCAACT | Sangon, China |
| M-IL-6 | F-GAGGATACCACTCCCAACAGACC  R-AAGTGCATCATCGTTGTTCATACA | Sangon, China |
| M-MCP-1 | F-CTCAGCCAGATGCAGTTAACGCCC  R-GGTGCTGAAGACCTTAGGGCAGAT | Sangon, China |
| M-β-Actin | F-CTGTCCCTGTATGCCTCTG  R-ATGTCACGCACGATTTCC | Sangon, China |
